# Vitamin D Deficiency and Oral Health: A Comprehensive Review

**DOI:** 10.3390/nu12051471

**Published:** 2020-05-19

**Authors:** João Botelho, Vanessa Machado, Luís Proença, Ana Sintra Delgado, José João Mendes

**Affiliations:** 1 Periodontology Department, Clinical Research Unit (CRU), Centro de Investigação Interdisciplinar Egas Moniz (CiiEM), Egas Moniz—Cooperativa de Ensino Superior, 2829-511 Caparica, Almada, Portugal; vmachado@egasmoniz.edu.pt; 2 CRU, CiiEM, Egas Moniz—Cooperativa de Ensino Superior, 2829-511 Caparica, Almada, Portugal; anasintradelgado@gmail.com (A.S.D.); jmendes@egasmoniz.edu.pt (J.J.M.); 3 Orthodontics Department, CRU, CiiEM, Egas Moniz–Cooperativa de Ensino Superior, 2829-511 Caparica, Almada, Portugal; 4 Quantitative Methods for Health Research Unit (MQIS), CiiEM, Egas Moniz—Cooperativa de Ensino Superior, 2829-511 Caparica, Almada, Portugal; lproenca@egasmoniz.edu.pt

**Keywords:** vitamins, vitamin D deficiency, oral health, periodontal health, periodontal disease, periodontitis, caries, orthodontics, oral cancer

## Abstract

Vitamin D (VD) levels have been gaining growing attention in Oral Health. During growth and adulthood, VD deficiency (VDD) is associated with a wide variety of oral health disorders, and impaired VD synthesis may expedite some of these conditions. In children, severe VDD can induce defective tooth mineralization, resulting in dentin and enamel defects. As a consequence, these defects may increase the risk of the onset and progression of dental caries. Further, VDD has been associated with higher prevalence of periodontitis and gingival inflammation, and several recent preclinical and clinical studies have unveiled potential pathways through which Vitamin D may interact with the periodontium. VDD correction through supplementation may contribute to a successful treatment of periodontitis; however, alveolar bone regeneration procedures performed in baseline VDD patients seem more prone to failure. Vitamin D may also be linked with some oral pathology entities such as certain oral cancers and events of osteonecrosis of the jaw. This review aims to provide comprehensive evidence of how VD levels should be considered to promote good oral health, and to summarize how VDD may hamper oral development and its role in certain oral conditions.

## 1. Introduction

**Figure 1:**
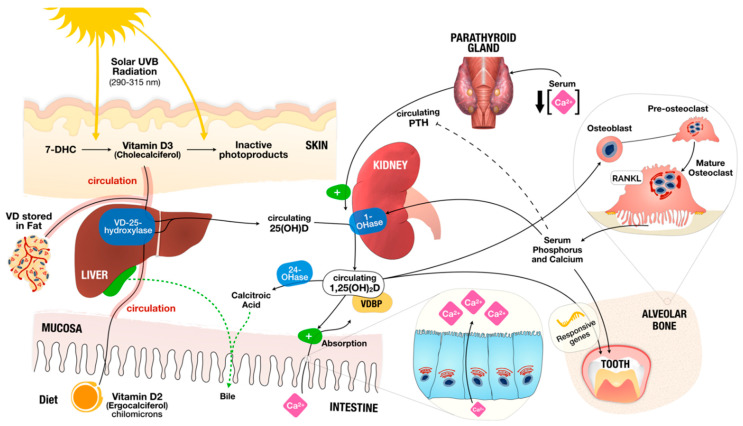
Vitamin D and Calcium, Phosphorus and Bone Metabolism. Vitamin D is obtained mostly from sunlight exposure (Vitamin D3) and from diet (Vitamin D2). In contact to solar ultraviolet B (UVB) radiation, 7-dehydrocholesterol (7-DHC) present in the skin is immediately converted to vitamin D3 in a heat-dependent process. Excessive sunlight exposure can destroy Vitamin D3 and convert it into inactive photoproducts. Vitamin D2 from diet is absorbed in the form of chylomicrons when they spread into the bloodstream as Vitamin D (encompassing Vitamin D2 or D3). Vitamin D in the circulation is bound to the vitamin D–binding protein (VDBP), which transports it to the liver. There, Vitamin D is converted by vitamin D-25-hydroxylase (VD-25-hydroxylase) to 25-hydroxyvitamin D (25(OH)D) (used as standard surrogate to determine vitamin D status). Then, 25(OH)D circulates, reaching the kidneys where it is activated by 25-hydroxyvitamin D-1αhydroxylase (1-OHase) to 1,25-dihydroxyvitamin D (1,25(OH)2D). Serum phosphorus or calcium can impact renal production of 1,25(OH)2D. 1,25(OH)2D promotes negative feedback of parathyroid hormone (PTH) by the parathyroid glands. 1,25(OH)2D increases the expression of 25-hydroxyvitamin D-24-hydroxylase (24-OHase), upholding its excretion in the bile. 1,25(OH)2D is recognized in the osteoblasts, causing the induction of mature osteoclast through expression of the receptor activator of nuclear factor-κB ligand (RANKL). Mature osteoclasts remove calcium and phosphorus from the bone, maintaining the serum levels of calcium and phosphorus.

Vitamin D is a steroid hormone obtained mainly from exposure to sunlight, but also from diet and dietary supplements [1,2,3,4,5]. Foods naturally containing vitamin D are rare, and it can be found in oily fish (such as salmon, mackerel, and herring) and oils from fish (e.g., cod liver oil) [2]. Vitamin D is a generic name comprising Vitamin D2 and D3. While Vitamin D2 is manufactured through ultraviolet irradiation of ergosterol from yeast, Vitamin D3 results from ultraviolet irradiation of 7-dehydrocholesterol from lanolin [4,6,7] (Figure 1) exhibiting the biological activity of cholecalciferol (vitamin D3), and it is synthesized in the human skin. Measurement of serum 25-hydroxyvitamin D (25[OH]D) is a widely accepted biomarker analysis for vitamin D status [3].

**Figure 2:**
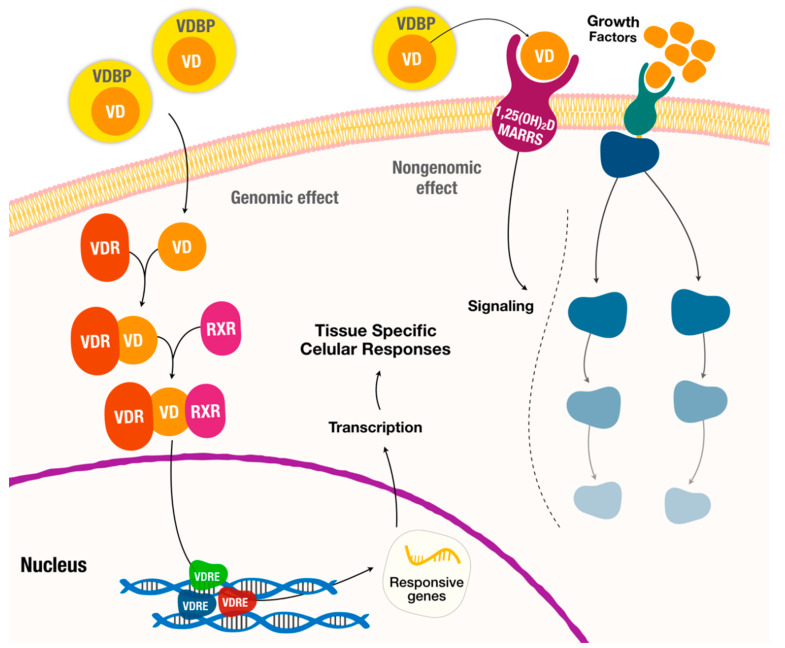
Hormonal actions of Vitamin D with genomic and nongenomic effects. MARRS-membrane-associated, rapid response steroid-binding protein; RDR—retinoid-X receptor; VD—Vitamin D; VDR—Vitamin D Receptor; VDRE—Vitamin D response elements; VDBP–Vitamin D-binding protein.

Vitamin D acts primordially as a hormone, and its endocrine activity promotes serum calcium and phosphate homeostasis through intestinal absorption regulation [2,6,8] (Figure 1). Vitamin D also acts like an autocrine and paracrine agent, regulating cell differentiation, cell maturation and innate immune system [9,10,11]. In detail, the cellular actions of Vitamin D are mediated through the Vitamin D receptor (VDR), which is a receptor molecule that binds to the active form of Vitamin D (Figure 2) [9,11,12,13]. As such, Vitamin D actions depend on the regulation of VDR for its genomic effects and on a membrane associated proteins to nongenomic effects (such as signaling pathways) [14] (Figure 2). This vast action is due to the fact that this vitamin modulates the expression of a considerable number of genes, and it is estimated to do so for 5–10% of the entire genome [9].

Public awareness of vitamin D has increased exponentially due to a worldwide prevalence of vitamin D deficiency (VDD) [2,3,7,15,16,17,18]. This prevalence is worrisome and is of the utmost importance for general health, with special focus on children, pregnancy, some forms of cancer and infection prevention [19,20,21,22].

In general, the major cause of VDD is the lack of exposure to sunlight with adequate ultraviolet B rays (exogenous factor) [6,17]. VDD can also arise from a nutritional deficit due to inadequate intakes of vitamin D, or hereditary disorders from absorption and metabolic conversion [6]. Additionally, drug related VDD is also possible due to iatrogenic increased clearance (for instance, with phenytoin, carbamazepine and oxcarbazepine regimens) [23].

The role of nutrition in oral diseases has gained popularity and recent investigations have uncovered increasingly relevant relationships between nutritional deficits and oral pathologies [24,25]. Concerning oral diseases, caries and periodontal diseases are complex multifactorial diseases and remain the two most prevalent diseases worldwide [26,27]. Both caries and periodontal disease are associated with VDD and its pathophysiologic processes [28,29,30,31]. The mechanisms by which vitamin D impacts oral health are not just based on bone metabolism. Nowadays, research has unveiled that VDD compromises odontogenesis, resulting in a hypomineralized dentition susceptible to fracture and caries lesions [29]. VDD was also linked to worse periodontal health and might be involved in the immune mechanism towards periodontal infection [32]. Furthermore, VDD in association with periodontitis has been recently linked to potential systemic repercussions during pregnancy, throughout orthodontic treatment, in post-menopausal women or in some oral pathologies [22,33,34].

The latest breakthroughs led us to elaborate a review aiming to comprehensively summarize the available evidence of the effect of vitamin D on oral health and its major complications. Furthermore, we discuss the impact of recent studies where VDD correction was implemented through supplementation which might underpin future clinical guidelines.

## 2. Vitamin D Deficiency Impact on Oral Health

### 2.1. Vitamin D Deficiency in Tooth Mineralization and Caries

Teeth are mineralized organs, surrounded by alveolar bone, and formed by three distinctive hard tissues: enamel, dentin, and cementum. The tooth mineralization process occurs parallel to skeletal mineralization, yet if mineral metabolism is disturbed then failures will occur similarly to those that occur in bone tissue. Vitamin D plays a key role in bone and tooth mineralization, and when levels are unregulated it can lead to the “rachitic tooth”, which is a defective and hypomineralized organ highly susceptible to fracture and decay [35,36].

The mechanisms by which VDD affects tooth mineralization are well debated elsewhere [35,36]. The main biological basis relies on the fact that severe VDD (<10 ng/mL) causes hypocalcemia and hypophosphatemia with secondary hyperparathyroidism (driven by hypocalcemia) [37,38]. This hyperparathyroidism promotes intestinal absorption of calcium (Ca^2+^), and renal production of 1 α,25-dihydroxyvitamin D (1,25[OH]2D), increasing bone turnover leading to elevated serum levels of Ca^2+^ and low serum levels of inorganic phosphate (Pi) [37,38]. The initial hypophosphatemia is then severely worsened. Ultimately, the loss of vitamin D signaling pathways in tooth cells with low concentrations of Ca^2+^ and phosphate ions inhibit proper mineralization of teeth and mineralization defects occur [35].

Apart from its mineralization homeostasis role, circulating vitamin D can initiate a signaling pathway through vitamin D receptors (VDR). VDR is a ligand-activated transcription factor that controls gene expression through vitamin D elements (VDRE) [39]. For instance, some of these responsive genes affect bone, mineral metabolism, immune response, cell life cycle and migration, skeletal muscle, detoxification, and energy metabolism [8,39,40,41,42,43]. Vitamin D upregulates VDR which, in turn, can induce structural gene products, including calcium-binding proteins and various extracellular matrix proteins (e.g., enamels, amelogenins, dentin sialoglycoproteins, and dentin phosphoproteins), resulting in the formation of dentin and enamel [35,44].

Beyond the typical VDD causes, nutritional deficiency or reduction of sunlight exposure, there are genetic deficiencies originating from mutations encoding elements of the vitamin D metabolic machinery. The main causes of VDD, second to genetic mutations, are abnormal enzyme secretion (i.e., vitamin D-dependent rickets type 1, VDDR-I) and anomalous VDR function or signaling (vitamin D-dependent rickets type 2, VDDR-IIa; hereditary defects in the vitamin D receptor-effector system, HDVDR) [35]. These genetic conditions cause defective mineralized tissues, despite normal vitamin D consumption or sunlight exposure and, ultimately, will increase the risk of mineralized tooth tissue hypoplasia (i.e., amelogenesis imperfecta, dentinogenesis imperfecta, enamel hypoplasia) or higher risk of caries [35].

Remarkably, deciduous dentition can be influenced by maternal 25(OH)D levels, despite the influence of inherited defects of the fetus [45,46]. Fetal serum-circulating levels of vitamin D follow the maternal concentration and can be used as a standard surrogate marker to the fetus [47]. Therefore, if maternal 25(OH)D levels turn unbalanced, this may have direct repercussions on the baby’s health [48] and, in particular, on tooth development [45,46,49,50,51]. The pattern of mineralization defect depends on the specific week of gestation when maternal VDD occurred. For example, approximately at the 13th week from conception, the human primary maxillary central incisor begins its calcification, and if there is a VDD status, there could be a hypoplasia/mineralization defect on the incisal third of the crown. Nowadays, it is known that maternal VDD at 12–16, 20–32 and 36–40 weeks results in defects at the incisal third, middle third and cervical third, respectively [51]. In a randomized clinical trial (RCT), vitamin D supplementation during pregnancy revealed that pregnant women with < 15 ng/mL of vitamin D had a 14% higher risk of deciduous dentition [51]. In contrast, high-dose vitamin D supplementation during pregnancy was associated with an approximately 50% reduced odds of enamel defects [49,52]. In another RCT, high-dose Vitamin D supplementation during pregnancy was linked to 50% lower risk of enamel defects in the newborn, underlying once more the likely preventive role of Vitamin D for enamel defects [49].

Furthermore, untreated caries in deciduous and permanent teeth were the most prevalent condition, affecting 9% and 35% of global population, respectively [53]. Moreover, according to WHO, caries is the fourth-most expensive chronic disease to treat [54]. This infectious disease has a complex and multifactorial etiology. Environmental factors, such as cariogenic diet with a high carbohydrate content, cariogenic bacteria, and poor oral hygiene were the most widely studied risk factors [55,56,57]. Nevertheless, when exposed to the same environmental risk factors, some patients are more susceptible or resistant to caries than others, so that environmental factors alone are insufficient to explain the prevalence and incidence of caries [58,59,60]. Currently the evidence highlights the association of low levels of vitamin D and the high prevalence of caries in both children and adults, although the mechanism remains unclear [61,62,63,64,65,66,67,68].

Additionally, vitamin D exerts several roles in the control of the human immune system, and an optimal vitamin D concentration (≥75 nmol/L) is associated with lower odds for dental caries in children [29,69,70]. However, the studies’ results are contradictory [45,62,71,72]. A recent systematic review of controlled clinical trials, with data from 2827 children, investigated the impact of vitamin D supplementation on dental caries prevention [28,73]. The results of this study show that vitamin D supplementation reduced the risk of caries in about 47%, but with low certainty [28]. Another research supports that caries-free children were twice as likely to have optimal vitamin D concentrations (≥75 nmol/L) and those with severe early childhood caries were at nearly three times the odds of having deficient levels (<35 nmol/L) [29]. On the one hand, it is important to clarify that serum vitamin D does not change the major structure of teeth since this structure remains constant until some extrinsic factor causes its wear. Notwithstanding, apparently vitamin D prevents caries lesions through immune regulation, promoting microbial eradication with peptide activity as discussed above.

The roles of both UVB and antimicrobial peptides (AMPs) in cariogenic bacteria reduction have been studied [74,75,76,77,78,79,80,81]. The mechanism through which UVB reduces the risk of dental caries is likely to be through the production of vitamin D and followed by the induction of AMPs, which have antimicrobial properties [74,76,77,78,79,80,81]. AMPs are host defense peptides, mostly cationic and amphiphilic molecules, that are essential elements of the innate immunity against several bacteria, fungi and viruses [75,78]. Investigations seem to point to a combination of AMPs rather than a specific role of a single AMP [79], and they have been proposed as potential application for the prevention and treatment of dental caries [80,81]. Remarkably, *Streptococcus mutans*, a primary etiological agent of dental caries, may resist host salivary AMPs explaining its virulence in dental caries pathophysiology [74,77,80].

Hence, we can conclude that vitamin D control levels prior to conception may be important to reduce the risk of enamel defects in deciduous teeth and should be controlled throughout pregnancy and after delivery.

### 2.2. Vitamin D Deficiency and Periodontitis

Periodontitis is a complex polymicrobial disease induced by plaque and with persistent chronic inflammation [82]. Periodontitis is one of the two most prevalent diseases worldwide and its severe stage is the sixth most prevalent, with strong socioeconomic and systemic repercussions [26,27,83,84,85]. It has great impact on quality of life and is recovered after periodontal therapy [86,87]. The systemic link between periodontitis and other diseases and conditions has escalated, such as diabetes [88], ischemic stroke [89], cardiovascular disease (CVD) [90], rheumatoid arthritis [91], inflammatory bowel disease [92], stress [93], solid-organ transplanted individuals [94], or preterm birth [95]. Furthermore, the impact of nutrition on periodontal health, and in particular VDD, has been intensively investigated [96,97,98,99,100] and a recent European consensus stated that an inadequate vitamin D status impacts periodontal health and oral functions [24].

Several cross-sectional studies have compared the levels of Vitamin D between individuals with periodontitis and without periodontitis; however, the results remain diverse. While most reports show that periodontitis was associated with lower levels of Vitamin D compared to non-periodontitis [101,102,103,104,105], another has reported no differences [106]. Further, vitamin D concentrations were associated with higher periodontal destruction, severe periodontitis stages and higher tooth loss [30,107,108,109]. In otherwise healthy patients (CVD and diabetes mellitus), lower levels of Vitamin D were also associated when periodontitis was diagnosed [102,104].

Data from the NHANES III study, performed in the USA, showed that individuals with the highest levels of vitamin D experienced 20% less bleeding on probing than those with the lowest levels [30]. Other investigations also demonstrated that lower levels of gingival inflammation are associated with people without periodontitis [101,104].

Comprehensively, the inflammatory and immune actions against periodontal pathogens are triggered by the host immune system. As previously mentioned, salivary low levels of vitamin D were associated with higher levels of inflammation biomarkers in periodontitis patients when compared to periodontally healthy patients (namely IL-35, IL-17A and transforming growth factor), supporting the presence of an inflammatory microenvironment [106]. Remarkably, vitamin D supplementation was linked to a decrease of salivary cytokines before nonsurgical periodontal treatment [110]. In addition, a cross-sectional study showed through gingival samples that periodontitis patients exhibited lower levels of VDR and fewer fibroblast cells with higher inflammatory cell infiltration compared with healthy periodontal individuals [111].

Although not fully understood, Vitamin D has apparent fine-tuning, anti-inflammatory and mineralization effects on the periodontium according to the latest in vitro evidence. A study showed that vitamin D may decrease the number of live porphyromonas gingivalis through active autophagy [112] and might alleviate the inflammatory burden of periodontitis in rodent models: decreasing inflammatory levels (RANKL, TNF-α, IL-1, MMP-9) [113,114,115,116]; inhibiting IL-6 overexpression [117]; and suppressing alveolar damage via inhibition of bone loss, apparently through systemic T-helper cells [118]. In cultured human periodontal cells, Vitamin D induced a comparable mineralization effect to vitamin C [119].

**Figure 3:**
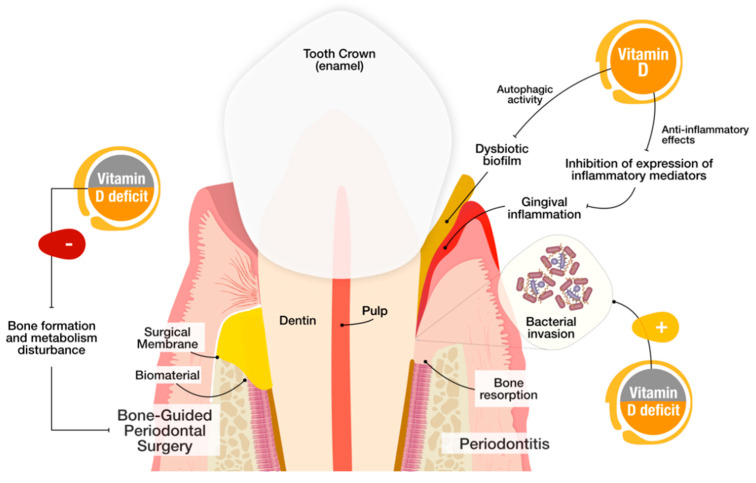
Suggested mechanisms of Vitamin D and Vitamin D deficiency (VDD) impact on periodontitis and periodontal regenerative surgery. (**Left**) Baseline VDD patients who undergo periodontal surgeries may be more prone to treatment failure due to the disturbance of bone formation and metabolism. (**Right**) In periodontitis, Vitamin D may promote *P. gingivalis* autophagy and anti-inflammatory effects via inhibition of expression of inflammatory mediators, decreasing gingival inflammation.

Thus both preclinical and clinical studies support the idea that vitamin D, through its metabolic pathway, might be involved in the pathogenesis of periodontitis, by impacting tooth mineral density and being reversely correlated with disease severity of periodontitis [25,97,120] (Figure 3).

From a genetic perspective, the role of VDR variants in periodontitis has been the subject of great consideration. In two recent evidence-based studies, a number of VDR polymorphisms were correlated with higher risk of developing periodontitis [121,122]. Notwithstanding, the VDR variants’ impact on periodontitis still remains to be consolidated since it depends on the number of studies, and is expected to increase considerably in the future.

On the other hand, the influence of vitamin D supplementation was studied in both nonsurgical and surgical periodontal treatments. Vitamin D and calcium supplementation showed moderate positive effects on periodontal health after nonsurgical periodontal treatment [98,110,111,123,124]. Further, baseline VDD negatively influenced periodontal surgery outcomes, even when supplementation was used to compensate for low levels [125] (Figure 3). Despite that these studies show vitamin D as a hypothetical hallmark for the success of patients’ treatment, more studies are warranted to infer scientific fallouts and permit definite conclusions.

In addition, a number of reports have explored the association between periodontitis and maternal VDD and found that pregnant women with moderate to severe periodontitis were linked to lower serum levels of vitamin D, compared to women with periodontal health [99,126,127,128,129]. Importantly, non-surgical periodontal treatment during pregnancy was proved to be successful in reducing adverse pregnancy outcomes; however, concomitant vitamin D supplementation showed mild clinical improvements in birthweight [128,130,131,132,133]. As such, future studies should assess this relationship in greater detail considering the amount of evidence that Vitamin D impacts periodontal health and maternal health.

### 2.3. Vitamin D Deficiency and Orthodontics

With the increase in importance of aesthetic requirements, facial micro- and macro-aesthetics and smile have become a priority for adolescents and adults [134,135]. Consequently, orthodontic treatments have become increasingly prevalent.

Tooth movement relies on the application of predetermined forces that cause mechanical stimuli with two simultaneous processes: (1) bone resorption on the pressure site, through osteoclastic activity; and (2) bone formation on the tension site, by osteoblastic action [136,137,138]. These two processes in combination with mechanical, chemical or electrical stimuli, might result in faster teeth movement [139,140,141,142]. Regarding the chemical factors, vitamin D might play a key role in tooth movement during orthodontic treatment and has shown promising results [33]. Albeit animal observational in nature, there is an increasing body of evidence showing that local application of vitamin D results in a faster tooth movement [136,143,144,145]. Notwithstanding, VDD in animal models causes a slower rate of tooth movement, and consequent treatment delay or complications [136,143,144]. In this sense, future studies in humans are needed to determine if VDD has a clinically significant effect on tooth movement and if vitamin D supplementation during orthodontic treatment, for instance in hypovitaminosis cases, improves the coupling of formation and resorption in alveolar bone remodeling during orthodontic tooth movement. 

### 2.4. Vitamin D Deficiency and Oral Pathology

Vitamin D might play an important function in the onset and progression of certain oral cancers, though there is still much to research. Remarkably, VDD is more common in patients with oral neoplastic lesions [22]. In a case-control study, VDD was associated with increased risk of squamous cell carcinoma of the esophagus, oral, and pharyngeal cancers, which were more prevalent in heavy smokers and severe alcoholism [146]. Another study has revealed that VDR expression was increased in premalignant lesions and oral cancer, and vitamin D supplementation significantly diminished therapy-related toxicities in late-stage oral cancers, with less morbidity and better quality of life [120]. Therefore, future studies should make efforts to clarify how VDD relates to oral cancer development and its therapeutic efficacy towards anticancer toxicity medications.

The role of VDD in osteonecrosis of the jaw (ONJ) is an increasingly discussed topic; however, Vitamin D status in ONJ individuals remains unestablished [147,148,149]. ONJ is characterized by a progressive death of the exposed jawbone in a patient chronically exposed to anti-absorption or anti-angiogenic medications, and no history of mandibular radiotherapy or metastatic disease of the jaw (Patel et al., 2018). Due to its role in bone mineralization, several studies have investigated the levels of Vitamin D in ONJ cases, mainly derived from osteoporosis and cancer patients [148,149,150,151,152,153,154]. The most recent evidence suggests that VDD is not a risk factor for the onset of ONJ events [148,154], while other studies underline a potential role [149,153]. Nonetheless, future studies should focus on the influence of baseline VDD and of Vitamin D supplementation towards ONJ, as well as the establishment of 25(OH)D level standards for patients at high risk of ONJ events.

## 3. Conclusions

VDD is highly implicated with oral diseases and has been linked with a higher risk of tooth defects, caries, periodontitis and oral treatments failure. The maintenance of appropriate 25(OH)D levels has shown to be associated with better oral development and health throughout life, though the impact of VDD correction through supplementation demands more evidence to allow definitive conclusions and potential clinical guidelines. Nevertheless, 25(OH)D levels should be considered to ensure a balanced oral health, and these levels need to be verified before the treatment of any oral conditions to warrant fruitful treatment outcomes.
